# Incidence and Mortality of Community‐Acquired Sepsis or Bacteraemia in Patients With Alcohol‐Related Cirrhosis

**DOI:** 10.1111/liv.70881

**Published:** 2026-09-15

**Authors:** Clara Juul Stenderup, Margarita Dudina, Kirstine Kobberøe Søgaard, Morten Daniel Jensen, Peter Jepsen

**Affiliations:** ^1^ Department of Hepatology and Gastroenterology Aarhus University Hospital Aarhus Denmark; ^2^ Department of Clinical Medicine Aarhus University Aarhus Denmark; ^3^ Department of Clinical Microbiology Aarhus University Hospital Aarhus Denmark; ^4^ Department of Clinical Microbiology Aalborg University Hospital Aalborg Denmark; ^5^ Department of Clinical Medicine Aalborg University Aalborg Denmark; ^6^ Department of Clinical Epidemiology Aarhus University Hospital Aarhus Denmark

**Keywords:** alcoholic cirrhosis, bacteremia, prognosis, sepsis

## Abstract

**Background & Aims:**

Liver cirrhosis is associated with an increased risk of bacterial infections, but there is a lack of reliable population‐based data to quantify this risk. We examined the incidence and predictors of community‐acquired serious systemic infections.

**Methods:**

This was a population‐based study that included all patients in the Central Denmark Region diagnosed with alcohol‐related cirrhosis (ALD cirrhosis) in 2013–2022. We computed the cumulative incidence of community‐acquired serious systemic infections, encompassing sepsis and bacteraemia. We used Fine & Gray regression to investigate predictors of infection within the first year.

**Results:**

We included 2511 patients with ALD cirrhosis (69.3% males, median age 61 years, median MELD‐NA score 13). Their cumulative risk of a first‐time community‐acquired serious systemic infection was 3.8% (95% CI: 3.1–4.6) after 1 year and 5.9% (95% CI: 5.0–6.9) after 3 years. The most frequent causative pathogens were Enterobacterales (38%), Staphylococci (25%) and Streptococci (22%). Increasing MELD‐Na score at cirrhosis diagnosis was associated with an increased risk of serious systemic infection within the first year after diagnosis (subdistribution hazard ratio = 1.05, 95% CI: 1.02–1.08, per 1 point increase). The 30‐day all‐cause mortality was 30% (95% CI: 23–37) following hospitalization with a serious systemic infection.

**Conclusions:**

This population‐based study determined the risk and mortality of serious systemic infections in ALD cirrhosis. Our findings will be important for future studies of interventions to prevent infections.

## Lay Summary/Key Points

We examined the risk of and mortality following bloodstream infections in Danish patients diagnosed with alcohol‐related liver cirrhosis in 2013–2022. We included 2511 patients, and their risk of bloodstream infection was 3.8% after 1 year and 5.9% after 3 years. Those with more advanced cirrhosis had a higher risk of infection. After infection, 30% of patients died within 30 days. Our findings will be important for future studies of interventions to prevent bloodstream infections.

**Abbreviations**
ACLF: acute‐on‐chronic liver failure
ALD cirrhosis: alcohol‐related liver disease cirrhosis
CPR number: Civil Personal Registration number
CRP: C‐reactive protein

*E. coli*

:
*Escherichia coli*

*E. faecalis*

:
*Enterococcus faecalis*

*E. faecium*

:
*Enterococcus faecium*

ICD‐10: International Classification of Diseases, Tenth Revision
INR: international normalized ratio
IQR: interquartile range
MELD‐Na: Model for End‐Stage Liver Disease score (with sodium)

*P. aeruginosa*

:
*Pseudomonas aeruginosa*

Ref.: reference
Ref. int.: reference interval

*S. aureus*

:
*Staphylococcus aureus*

SHR: subdistribution hazard ratio
Spp.: species
TOKS: Danish: ‘Tidlig Opsporing af Kritisk Sygdom’. English translation: ‘Early Detection of Critical Illness’

## 1 Introduction

Patients with alcohol‐related cirrhosis (ALD cirrhosis) are at increased risk of bacterial infections [1, 2, 3], and serious systemic infections such as sepsis or bacteraemia can be fatal. Specifically, sepsis is the cause of acute‐on‐chronic liver failure (ACLF) in up to 30% of cases [4, 5], but the incidence of these serious systemic infections among cirrhosis patients remains unknown.

A Swedish study focused on hospital‐diagnosed infections in patients with alcohol‐related liver disease of any stage (59% cirrhosis) and found an increased risk in patients compared with population controls [6]. Similarly, a Danish study, also in patients with alcohol‐related liver disease of any stage (only 14% with cirrhosis), found a 1‐year risk of severe infections of approximately 4% [7]. A recent Chinese meta‐analysis [8] found that *Escherichia coli* , Klebsiella, Staphylococci and Streptococci were among the most prevalent bacteria found in positive blood cultures from infected cirrhosis patients worldwide. Further, they reported a 30‐day mortality of 26% following hospitalization with any serious infection. However, existing studies distinguishing between community‐acquired and hospital‐acquired infections are yet sparse. Accordingly, there is a gap in our understanding of the risk of community‐acquired serious systemic infections in ALD cirrhosis to aid optimising preventive measures [1, 9, 10]. Information from a more homogenous study population is valuable for patients and their clinicians, and it is crucial for designers of experiments: Preventive measures, such as vaccines, antibiotics, or faecal microbiota transplantation, may decrease the risk of serious systemic infections in cirrhosis patients, but randomized trials of preventive measures need accurate estimates of the incidence of infections for their sample size estimations [11].

With infections being a well‐known threat to all cirrhosis patients, a more comprehensive epidemiological characterization of bacterial infections in cirrhosis is needed—both on a national and a global geographical level. We aimed to estimate the incidence of community‐acquired serious infections (defined as sepsis or bacteraemia) in a cohort of Danish patients with ALD cirrhosis. We described the microbiological aetiology, examined potential predictors of infection, and the mortality associated with these serious infections in patients with ALD cirrhosis.

## 2 Methods

### 2.1 Data Source

We used data covering all citizens from the Central Denmark Region, a population of 1.36 million [12] (the total Danish population was 5.9 million in 2023 [13]). Data were extracted directly from the regional electronic health record system ‘MidtEPJ’ used by all hospitals in the Central Denmark Region since 2012. Patients were identified in the system by their Civil Personal Registration number (CPR number), which all Danish citizens are assigned at birth or immigration [14]. The dataset contained information on all in‐ and outpatient hospital contacts including data on diagnoses, interventions, treatments, microbiological examinations, biochemistry, comorbidities and medication. Diagnoses were coded using the International Classification of Diseases, 10th Revision (ICD‐10), and we considered both primary and secondary diagnoses. The primary diagnosis code denotes the condition responsible for the hospital contact, whilst secondary diagnosis codes describe other relevant diseases or conditions. According to Danish law, approval for registry‐based studies is granted by the patients or the administrative region rather than the Ethics Committee. The project was approved by the Central Denmark Region, and the requirement for patient consent was waived due to a large cohort and a large proportion of the patients being deceased (case number 1‐45‐70‐47‐23).

### 2.2 Study Cohort

We identified all adult (≥ 18 years) citizens in the Central Denmark region who were diagnosed with ALD cirrhosis between 1 January 2013 and 1 September 2023. Patients were identified by the ICD‐10 codes K70.3 (ALD cirrhosis) and K70.4 (alcohol‐related liver failure). They received their diagnosis code from the attending physician upon completion of a hospital contact (e.g., at discharge from admission or at the outpatient visit). Patients who had experienced a serious systemic infection (as defined below) within 90 days before the inclusion date were excluded from the study cohort, the objective being to compute the incidence of first‐time infection following cirrhosis diagnosis without including ongoing/prevalent infections. As a results, 13 patients were excluded.

### 2.3 Study Design

The patients were included on the admission date of the first hospital contact with an ALD cirrhosis diagnosis within the study period. They were followed from inclusion until either admission with a community‐acquired serious systemic infection (the outcome), a hospital‐acquired serious systemic infection (competing risk event), death (competing risk event), or end of follow‐up on 1 September 2023 (administrative censoring), whichever came first. The subgroup of patients who experienced a community‐acquired serious systemic infection was additionally followed from the date of admission with that infection until death (the outcome) or end of follow‐up (administrative censoring) to assess all‐cause mortality. The study design is illustrated in Figure S1. During the follow‐up, 11 patients received a liver transplant; this was not considered a competing risk event.

### 2.4 Serious Systemic Infections

Our primary outcome was a first‐time hospitalization with a community‐acquired serious systemic infection. This outcome was defined by a positive blood culture and/or a diagnosis code indicating sepsis or bacteraemia to improve sensitivity of the outcome.

#### 2.4.1 Identification of Serious Systemic Infections

First, a clinical microbiologist (coauthor M.D.) reviewed all positive blood cultures and excluded obvious skin contaminations (e.g., coagulase‐negative Staphylococci, *Micrococcus luteus* or coryneform bacteria when present in only 1 blood culture bottle). As a standard procedure, two sets of blood cultures were collected from each patient, each set consisting of one aerobic and one anaerobic bottle. Blood cultures were processed using the Bact/ALERT system (BioMérieux, Marcy‐l'Étoile, France). Bacteria were identified using conventional biochemical tests and matrix‐assisted laser desorption/ionization time‐of‐flight (MALDI‐TOF) mass spectrometry (Bruker, Bremen, Germany).

Second, we considered discharge diagnosis codes for sepsis and bacteraemia and defined two groups (sepsis and bacteraemia) that were combined under the umbrella term ‘serious systemic infections’ (diagnosis codes are listed in Table S1). By our definition, patients with sepsis had a diagnosis code for sepsis with or without a positive blood culture, and patients with bacteraemia had a positive blood culture with or without a diagnosis code for bacteraemia, but without a diagnosis code for sepsis. These definitions failed to classify one patient, who had a diagnosis code for bacteraemia but no positive blood culture from that hospitalization; we chose to exclude this patient.

Third, having identified the serious systemic infections, we divided them into community‐acquired and hospital‐acquired infections. Community‐acquired infections covered infections in patients admitted from home with no recent hospitalization (within 6 days prior to the admission) [15]. In patients with a positive blood culture, the infection was defined as community‐acquired if the blood culture was sampled within 48 h of admission. In this case, the date of infection was set as the date the blood sample was drawn. If the infection was identified based only on a discharge diagnosis code, we used clinical data to corroborate the diagnosis and classify the infection as community‐ or hospital‐acquired. It was classified as community‐acquired if at least one of these two criteria were met: (1) blood cultures were taken within 48 h of admission (even if they came back negative); (2) within 48 h of admission, the patient had a C‐reactive protein (CRP) measurement over 100 mg/L and a so‐called ‘TOKS score’ of at least 5. A TOKS score of 5 indicates that the patient must immediately be consulted with the attending senior doctor [16]. TOKS is a mandatory critical illness early warning system for inpatients in the Central Denmark Region, similarly to what is recommended in England [17]. All serious systemic infections that did not meet the criteria for being community‐acquired were defined as hospital‐acquired.

### 2.5 Biochemistry

Measurements of bilirubin, albumin, platelets, INR, sodium and creatinine were used to describe the severity of the patients' liver cirrhosis at the time of the cirrhosis diagnosis. We extracted the measurements closest to the date when the patients were first diagnosed with ALD cirrhosis. If no measurements were available from the date of diagnosis, we allowed measures from up to 7 days before. If still no measurements were available, measurements from the day after diagnosis were included. If more than one measurement was available from the same date, the highest value was used for bilirubin, INR and creatinine, and the lowest for albumin, platelets and sodium. MELD‐Na score (Model for End‐Stage Liver Disease‐Sodium) was calculated when all relevant biochemical measurements were available [18].

### 2.6 Mortality After Serious Systemic Infection

Among the subcohort of patients who experienced a community‐acquired serious systemic infection we computed all‐cause mortality after infection. Follow‐up began on the date of the serious systemic infection and patients were followed until death or censoring at the end of follow‐up on 1 September 2023.

### 2.7 Statistical Analyses

#### 2.7.1 Incidence of Community‐Acquired Serious Systemic Infections

We computed the cumulative risk of first‐time admission with community‐acquired serious systemic infection using the cumulative incidence function, with death and hospital‐acquired serious systemic infections as competing risks. Further, we reported the incidence rate as the number of first‐time serious systemic infections divided by the total observation time at risk. Additionally, we reported the distribution of the bacterial agents identified through blood cultures.

#### 2.7.2 Predictors for Community‐Acquired Serious Systemic Infections

We used a Fine & Gray regression model to identify predictors associated with a high risk of community‐acquired serious systemic infections, considering death and hospital‐acquired serious systemic infections as competing risks. We performed both a univariable and a multivariable analysis. The univariable regression model considered the patient's age, sex, MELD‐Na score, bilirubin, albumin, platelets, INR, sodium and creatinine. We also investigated the association between these factors and the risk of death without community‐acquired serious systemic infection. The multivariable model included age, sex and MELD‐Na score. For both analyses, follow‐up ended 1 year after ALD cirrhosis diagnosis. The analyses were performed as complete‐case analyses, meaning that patients with missing values were excluded.

#### 2.7.3 Sensitivity Analysis

We repeated the analysis of incidence of serious systemic infection analysis altering follow‐up to start at 30 days after patients were diagnosed with ALD cirrhosis, only including patients alive at that point in time. The purpose was to restrict to a cohort where preventive interventions theoretically could have been offered when cirrhosis was diagnosed.

#### 2.7.4 Mortality After Serious Systemic Infections

In patients who experienced a community‐acquired serious systemic infection, we computed all‐cause mortality risk following the infection using the Kaplan–Meier estimator. Additionally, we calculated mortality risk after sepsis and bacteraemia separately in order to compare the two groups and assess the decision to combine them.

All analyses were carried out using Stata version 18.5.

## 3 Results

We included 2511 patients (30.7% females) with ALD cirrhosis, and their median age was 61 years (IQR 54–68) at the time of diagnosis (Table 1). At inclusion, the study cohort was characterized by hypoalbuminaemia (median 26 g/L, IQR 21–31), hyperbilirubinaemia (median 36 μmol/L, IQR 18–73), elevated INR (median 1.5, IQR 1.3–1.8) and low platelet count (median 131 × 10^9^/L, IQR 84–201). However, full biochemistry was not available for the entire cohort. The MELD‐Na score was available for 47% (*N* = 1189) of the cohort, and the median MELD‐Na score for these patients was 13 (IQR 10–21) (Table 1).

**TABLE 1:** Baseline patient characteristics at inclusion, *N* = 2511.

	Median (IQR)^b^	Missing values, *N* (%)
Sex, no. female (%)	772 (30.7%)	0 (0%)
Age, median (IQR)	61.3 (54.3–68.3)	0 (0%)
Albumin (Ref. int. 36–45)	26 (21–31)	844 (34%)
Bilirubin (Ref. int. 5–25)	36 (18–73)	882 (35%)
INR (Ref. int. < 1.2)	1.5 (1.3–1.8)	1223 (49%)
Creatinine (Ref. int. female 45–90, male 60–105)	68 (52–97)	741 (30%)
Platelets (Ref. int. female 165–400, male 145–350)	131 (84–201)	1042 (41.5%)
Sodium (Na) (Ref. int. 137–145)	136 (132–139)	766 (30.5%)
MELD‐Na score^a^	13 (10–22)	1322 (53%)

*Note:* Unit of blood measurements: Albumin, plasma g/L; bilirubin plasma μmol/L; INR, plasma; creatinine, plasma μmol/L; platelets, whole blood ×10^9^/L; sodium, plasma mmol/L. Reference intervals as determined by Department of Clinical Biochemistry, Aarhus University Hospital [19].

Abbreviations: IQR, interquartile range; ref. int., reference interval.

^a^ MELD score calculated according to HRSA guidelines [18].

^b^ All measures are median with IQR, except sex which shows the number of females and the corresponding proportion.

### 3.1 Incidence of Community‐Acquired Serious Systemic Infections

**FIGURE 1:**
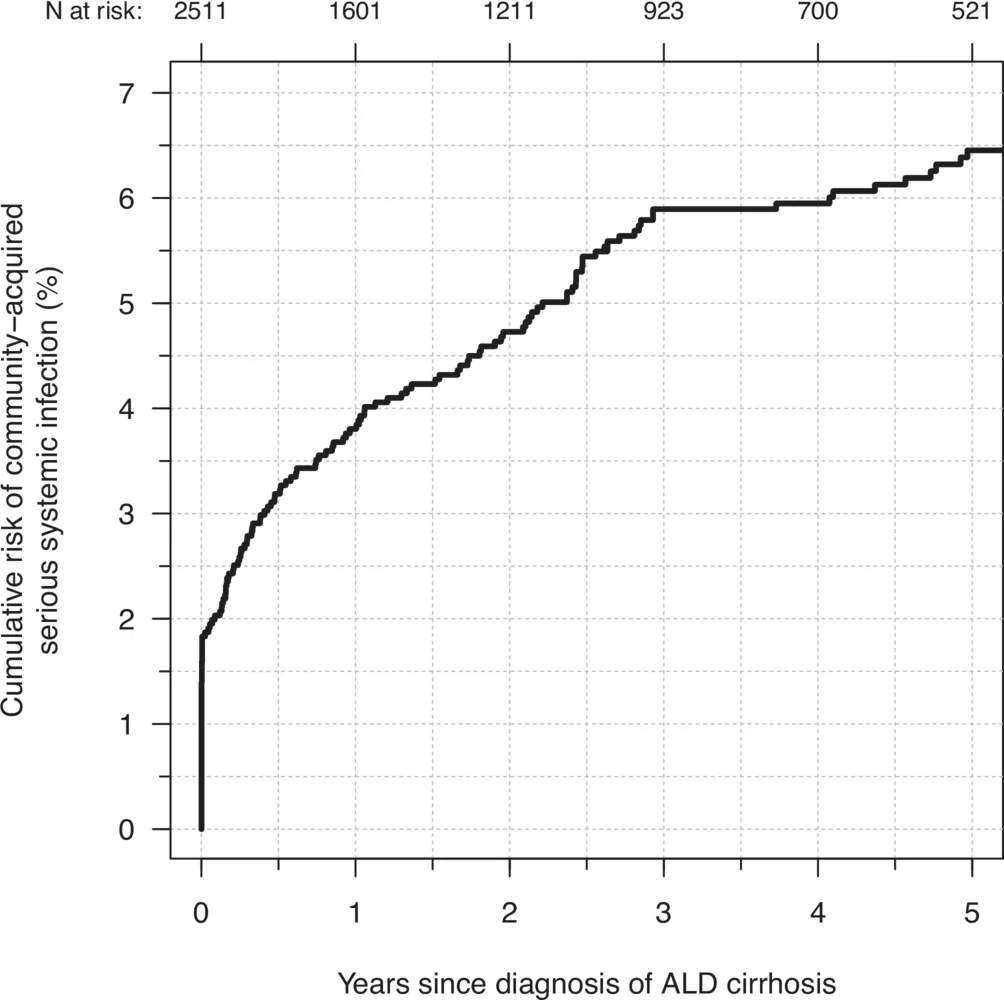
Cumulative risk of community‐acquired serious systemic infection, *N* = 2511. One hundred and fifty‐six patients total experienced a community‐acquired serious systemic infection. Death and hospital‐acquired serious systemic infections were treated as competing risks.

The cumulative risk of a community‐acquired serious systemic infection was: 30‐day, 2.0% (95% CI: 1.5–2.6); 90‐day, 2.6% (95% CI: 2.0–3.2); 1‐year, 3.8% (95% CI: 3.1–4.6); and 3‐year, 5.9% (95% CI: 5.0–6.9) (Figure 1). During the total 7024 years of follow‐up, the incidence rate of a first‐time community‐acquired serious systemic infection was 22.2 per 1000 person‐years (95% CI: 18.9–25.9). A total of 156 patients experienced a first‐time hospitalization with a community‐acquired serious systemic infection following diagnosis of ALD cirrhosis; among them, 42 (26.9%) infections were identified by a diagnosis code, 63 (40.4%) were identified only by a positive bacterial blood‐culture, whilst 51 (32.7%) infections were identified by both. Meanwhile, 302 patients experienced a hospital‐acquired serious systemic infection.

#### 3.1.1 Distribution of Bacterial Agents

In total, 114 patients had a positive blood culture during follow‐up. For 12 patients, the bacteraemia was polymicrobial with up to seven different species in one blood culture set. Among the 114 patients, a total of 133 different species were identified: Gram‐positive bacteria (*N* = 70, 53%), Gram‐negative bacteria (*N* = 53, 40%) and anaerobic bacteria (*N* = 10, 7%). The most frequent bacteria were Enterobacterales (*N* = 51, 38%), Staphylococci (*N* = 33, 25%) and Streptococci (*N* = 29, 22%). *Escherichia coli* constituted 59% (*N* = 30) of the Enterobacterales, whereas *S. aureus* constituted 91% (*N* = 30) of the Staphylococci. Pneumococci constituted only 3% of all culture‐positive bacteraemia (*N* = 4), fewer than β‐haemolytic (*N* = 14, 11%) and non‐haemolytic (*N* = 11, 8%) streptococci. See Table 2 for a full mapping of bacteria found in patients with a positive blood culture.

**TABLE 2:** Distribution of bacterial agents.

Classification	No. (%)	Classification	No. (%)	Classification	No. (%)
Gram‐positive	70 (53%)	*Staphylococcus*	33 (25%)	*S. aureus*	30 (22.5%)
				Coagulase‐negative staphylococci	3 (2%)
		*Streptococcus*	29 (22%)	Pneumococci	4 (3%)
				Β‐haemolytic streptococci	14 (11%)
				Non‐haemolytic streptococci	11 (8%)
		*Enterococcus*	6 (4.5%)	*E. faecalis*	4 (3%)
				*E. faecium*	1 (0.75%)
				Other Enterococci	1 (0.75%)
Gram‐negative	53 (40%)	Enterobacterales	51 (38%)	*Escherichia coli*	30 (23%)
				Non‐ *E. coli*	21 (16%)
		Aerobic gram‐negative rods	1 (0.75%)	Non‐ *Pseudomonas aeruginosa*	1 (0.75%)
		Gram‐negative rods	1 (0.75%)	Unknown	1 (0.75%)
Anaerobic	10 (7%)	Gram‐positive anaerobic	5 (3.75%)	*Clostridium* spp.	3 (2%)
				Other Gram‐positive anaerobic	2 (1.50%)
		Gram‐negative anaerobic	5 (3.75%)	*Bacteroides* spp.	2 (1.50%)
				Other Gram‐negative anaerobic	3 (2%)
Others		Others	2 (1.5%)	Others	2 (1.50%)
Total	133 (100%)		133 (100%)		133 (100%)

*Note:* Positive microbiology cultured in 114 patients with alcohol‐related (ALD) cirrhosis and community‐acquired serious systemic infections identified by either a positive culture (*N* = 63), or both a positive culture and a diagnosis code (*N* = 51). The bacterial agents were generally not specified for those infections identified solely by a diagnosis code. As 12 patients were identified with more than one infectious agent, the total number of bacteria (133) was higher than the number of patients with a positive culture.

#### 3.1.2 Predictors for Community‐Acquired Serious Systemic Infection

We found that the risk of a community‐acquired serious systemic infection within the first year after cirrhosis diagnosis increased with the MELD‐Na score at baseline, with a subdistribution hazard ratio (SHR) of 1.05 (95% CI: 1.02–1.08) per point increase. This effect persisted after adjustment for sex and age (Tables 3 and 4). Overall, markers of hepatic decompensation—high MELD‐Na score, low albumin, high bilirubin, high INR, low platelets and low sodium—were more strongly predictive of mortality than of a high risk of serious systemic infection (Table 3). The same pattern was true for older age and male sex, giving the overall impression that it is more difficult to predict who will have a serious systemic infection than who will die without such an infection.

**TABLE 3:** Univariable Fine & Gray regression models, one for each possible outcome.

Covariates	No.	Community‐acquired serious systemic infection^b^	Death without community‐acquired serious systemic infection^c^
		SHR^a^ (95% CI)	SHR^a^ (95% CI)
Age at inclusion (per 10‐year increase)	2511	1.10 (0.90–1.34)	**1.41 (1.28–1.55)**
Male sex (reference: female sex)	2511	0.87 (0.57–1.33)	1.28 (0.98–1.43)
MELD‐Na score (per 1 point increase)	1189	**1.05 (1.02–1.08)**	**1.06 (1.04–1.07)**
Albumin (per 5 g/L increase)	1667	**0.96 (0.93–0.99)**	**0.94 (0.92–0.95)**
Bilirubin (per 10 μmol/L increase)	1629	1.00 (0.98–1.02)	**1.03 (1.02–1.04)**
INR (per 0.1 increase)	1288	1.01 (0.99–1.04)	**1.05 (1.04–1.07)**
Creatinine (per 10 μmol/L increase)	1770	**1.03 (1.02–1.05)**	**1.04 (1.02–1.05)**
Platelets (per 10 × 10^9^/L increase)	1469	1.00 (0.99–1.00)	0.99 (0.99–1.00)
Sodium (per 5 mmol/L increase)	1745	0.99 (0.96–1.03)	**0.98 (0.97–0.99)**

*Note:* The values in bold are statistically significant results (*p* ≤ 0.05).

^a^ SHR = subdistribution hazard ratio estimated using Fine & Gray regression. Values represent the effect of each covariate on the cumulative incidence of the two respective outcomes within 1 year, accounting for competing risks. All covariates were measured as close as possible to date of cirrhosis diagnosis (see Section 2).

^b^ With death and hospital‐acquired systemic infection as competing risks.

^c^ With community‐acquired and hospital‐acquired systemic infection as competing risks.

**TABLE 4:** Multivariable Fine & Gray regression model.

Covariates	No.	Community‐acquired serious systemic infection^b^
		SHR^a^ (95% CI)
Age at inclusion (per 10‐year increase)	2511	1.07 (0.84–1.37)
Male sex (reference: female sex)	2511	0.93 (0.54–1.59)
MELD‐Na score (per 1 point increase)	1189	**1.05 (1.02–1.08)**

*Note:* The values in bold are statistically significant results (*p* ≤ 0.05).

^a^ SHR = subdistribution hazard ratio estimated using Fine & Gray regression. Values represent the effect of each covariate on the cumulative incidence of the outcome within 1 year, accounting for competing risks. All covariates were measured as close as possible to date of cirrhosis diagnosis (see Section 2).

^b^ With death and hospital‐acquired systemic infection as competing risks.

### 3.2 Sensitivity Analysis

**FIGURE 2:**
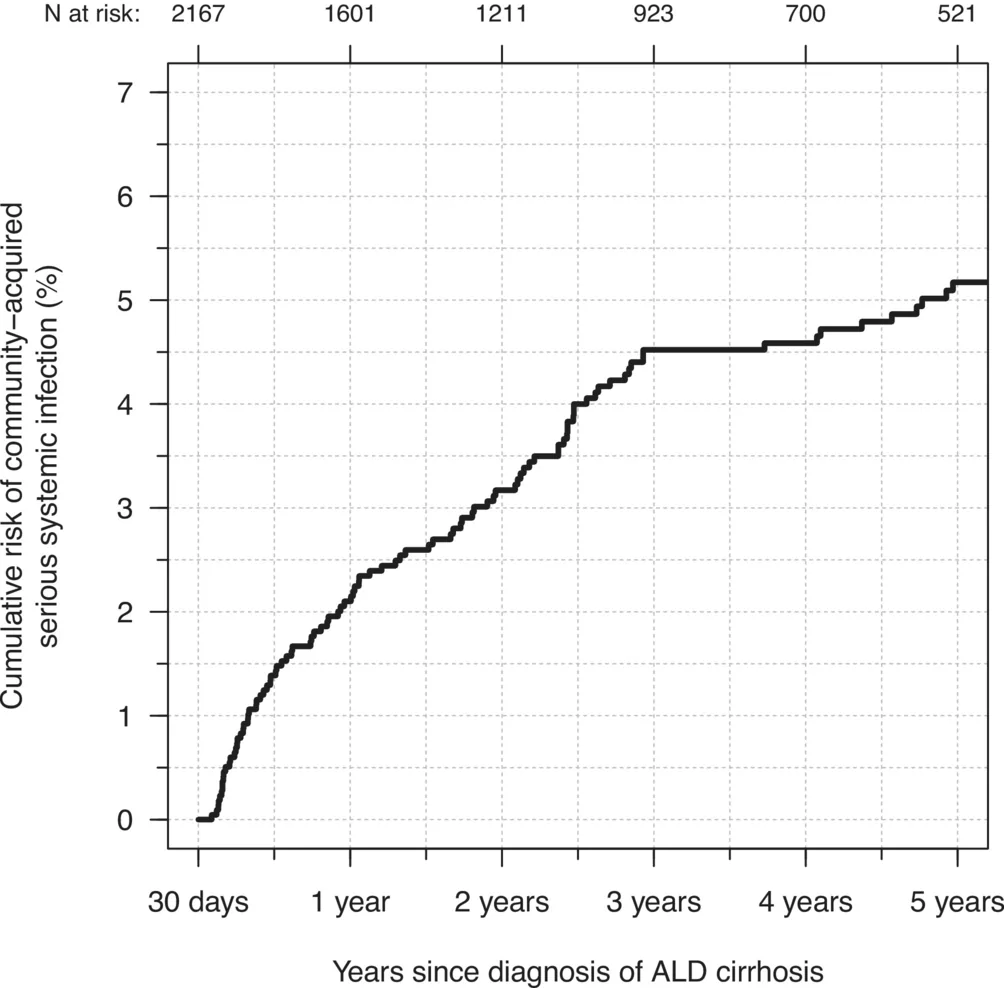
Cumulative risk of community‐acquired serious systemic infection, follow‐up start at 30 days after alcohol‐related (ALD) cirrhosis diagnosis, *N* = 2167. Death and hospital‐acquired serious systemic infections were treated as competing risks.

When starting follow‐up 30 days after the diagnosis of ALD cirrhosis, 2167 patients were available for inclusion. Among them, the 1‐year cumulative risk of a serious systemic infection was 2.3% (95% CI: 1.8–3.1) (Figure 2), and during a total of 6837 years of follow‐up, the incidence rate was 15.5 per 1000 person‐years (95% CI: 12.8–18.8) compared with 22.2 per 1000 person‐years in the primary analysis.

### 3.3 Mortality After Community‐Acquired Serious Systemic Infection

**FIGURE 3:**
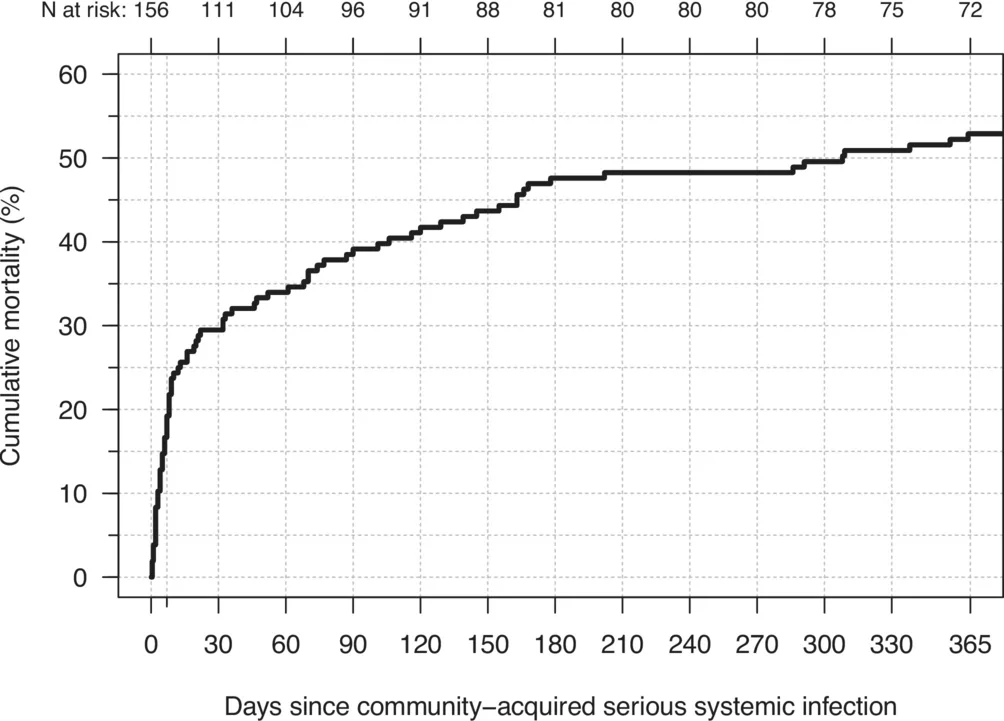
1‐year mortality after a community‐acquired serious systemic infection, *N* = 156.

We followed all 156 patients with ALD cirrhosis and a subsequent community‐acquired serious systemic infection to examine their all‐cause mortality risk following the serious systemic infection (Table 5). Among these patients, the 7‐day mortality risk was 19% (95% CI: 13.9–26.3), the 30‐day mortality risk was 30% (95% CI: 23.0–37.3) and the 90‐day mortality risk was 39% (95% CI: 32.0–47.3) (Figure 3). Stratifying the 90‐day mortality risk in patients with sepsis versus those with bacteraemia revealed no statistically significant difference (log‐rank test, *p* = 0.2) in mortality following infection (Figure S2).

**TABLE 5:** Characteristics of the infected subcohort at the time of infection, *N* = 156.

	Median (IQR)^b^	Missing values, *N* (%)
Sex, no. female (%)	49 (31.4%)	0 (0%)
Age, median (IQR)	62.5 (56.7–69.7)	0 (0%)
Albumin (Ref. int. 36–45)	24 (20–28)	6 (4%)
Bilirubin (Ref. int. 5–25)	42 (22–73)	7 (4.5%)
INR (Ref. int. < 1.2)	1.7 (1.3–2.0)	22 (14%)
Creatinine (Ref. int. female 45–90, male 60–105)	101 (67–209)	0 (0%)
Platelets (Ref. int. female 165–400, male 145–350)	107 (67–168)	6 (4%)
Sodium (Na) (Ref. int. 137–145)	134 (128–137)	4 (3%)
MELD‐Na score^a^	22 (14–27)	24 (15.5%)

*Note:* Unit of blood measurements: Albumin, plasma g/L; bilirubin plasma μmol/L; INR, plasma; creatinine, plasma μmol/L; platelets, whole blood ×10^9^/L; sodium, plasma mmol/L. Reference intervals as determined by Department of Clinical Biochemistry, Aarhus University Hospital [19].

Abbreviations: IQR, interquartile range; ref. int., reference interval.

^a^ MELD score calculated according to HRSA guidelines [18].

^b^ All measures are median with IQR, except sex which shows the number of females and the corresponding proportion.

## 4 Discussion

In our study of 2511 patients with ALD cirrhosis, we found a 1‐year risk of 3.8% for community‐acquired serious systemic infections leading to hospital admission, whereas the 5‐year risk was 6.5%. The most frequent causative pathogens were Enterobacterales (38%), Staphylococci (25%) and Streptococci (22%). Increasing MELD‐Na score at the time of diagnosis was associated with an increased risk of serious systemic infection within the first year after diagnosis. The mortality risk for patients with ALD cirrhosis and a serious systemic infection was 30% within 30 days after hospitalization with the serious systemic infection.

An important strength of this study was the use of the population‐based Central Denmark Region registry to identify all adult patients diagnosed with ALD cirrhosis in the Central Denmark Region [20]. Due to the structure of the Danish healthcare system and its handling of all cirrhosis patients at the regional hospitals, this study population is likely to be representative of ALD cirrhosis patients in all regions of Denmark, meaning that a high generalizability can be assumed [12]. We chose to focus this study on patients with cirrhosis attributable to alcohol as to acquire a more homogenous study cohort recognizable to clinicians. The disadvantage of this decision was that our results should not be generalized to patients with cirrhosis from other aetiologies.

Another strength of this study was the inclusion of microbiology data. Systemic bacterial infections are ideally diagnosed based on a positive blood culture, but some patients may not have a blood culture drawn at admission or may have negative blood cultures due to antibiotics prior to sampling. Thus, we would underestimate the risk of systemic infection if we required a positive blood culture. However, we would also underestimate the risk of systemic infection if we required a discharge diagnosis code for infection, because they are incompletely recorded [21, 22]. Consequently, we aimed to combine these approaches to increase the validity of our findings. As it is difficult to differentiate between ongoing and new infections following cirrhosis diagnosis, we excluded patients who had hospital contacts with sepsis or bacteraemia within 90 days of cirrhosis diagnosis. Accordingly, we attempted to increase the validity of the infections being community‐acquired by not allowing any sort of hospitalization 6 days prior to admission with serious systemic infection. At last, the use of CRP and TOKS increased the likelihood of the patients truly being critically ill with a community‐acquired serious systemic infection. Information on contacts with other parts of the Danish health care sector in the period before diagnosis would have been useful in confirming community‐acquired genesis; however, healthcare institutions of the primary and tertiary sector mainly use separate electronic health records systems.

By using microbiology data, we were also able to investigate the responsible bacterial agents. The most prevalent bacteria were found to be *E. coli* and *Staphylococcus aureus* . Interestingly, these bacteria are also the most prevalent pathogens in positive blood cultures in the general Danish population [23]. Mapping out the primary infection foci of the systemic infections found in this study would have been important clinical information for prevention studies, but unfortunately this data were incomplete. We encourage future studies to include information on primary infection foci, as to be able to target prophylactic measures more specifically.

However, there are also potential limitations in combining microbiology results and diagnosis codes for sepsis as *one* variable when identifying serious systemic infections. One could argue that grouping microbiological findings with a well‐defined clinical syndrome could pose a problem, considering that they do not represent the same disease entity. We argue that the decision to equate bacteraemia with sepsis was appropriate in this study, as bacteraemia and sepsis were shown to be of comparable fatality to the patients in the cohort (see Figure S2). Further, prophylactic measures targeting bacteria may potentially prevent both sepsis and bacteraemia, in turn supporting the clinical relevance of the grouping. Finally, the overall purpose of the study was to enable future studies on preventive measures to decrease the risk of any kind of serious systemic infection in ALD cirrhosis patients.

Another potential limitation was the validity of the diagnosis codes used to identify our cohort of patients with ALD cirrhosis [22]. Baseline data of our cohort from the time of diagnosis (Table 1) does however reflect a typical cohort of cirrhosis patients [24], indicating that the study population reliably represents a typical cohort of Danish ALD cirrhosis patients. Alcohol overuse is known to have an impact on infection rate, but a limitation to this study was the incompleteness of our data on alcohol use. We had access to standardized forms with categorical data on alcohol consumption, but this information was only recorded for 341 of the patients (14%). Among them, 219 (64%) had a current overuse at the time of cirrhosis diagnosis, 114 (33%) had a former overuse, whilst 8 (2%) allegedly never had any overuse of alcohol.

This study focused on ALD cirrhosis patients and community‐acquired serious systemic infections. This has not been examined before, but adds to the findings of two previous studies from Sweden [6] and Denmark [7]. Both studies included patients with different stages of alcohol‐related liver disease, but while the Swedish study included 4028 patients with alcohol‐related liver disease of whom 59% had cirrhosis (2370 patients), the Danish study included only 461 patients of whom 14% had cirrhosis (66 patients). The Swedish study focused on hospital‐diagnosed infections and found an increased risk of infections overall in patients compared with population controls, and as well for sepsis separately. Further, they found an increased mortality risk after a hospital‐based infection in patients compared with controls [6]. The Danish study found a 1‐year risk of severe infections of approximately 4% among all patients with alcohol‐related liver disease, and mortality was higher in patients with vs. without an infection. Our findings were compatible with the findings of these studies and offer an important addition by focusing on community‐acquired infections to aid in future optimisation of preventive clinical practice.

A recent Chinese meta‐analysis [8] from 2026 sought to characterize worldwide bacterial infections, microbiological profiles and mortality in cirrhosis based on 169 studies (published between 1982 and 2024). Forty‐five cohorts consisted solely of ALD cirrhosis patients. Based on 55 of all cohorts (31 European) they found that 1543 out of 12 515 infections were bacteraemia (10.5%). Only approximately half of the infections in 106 cohorts were confirmed by a positive microbiological culture. The pathogens found in our study corresponded well with the general findings of this study, with *E. coli* , Enterococci, Klebsiella and Staphylococci being among the most prevalent. The study attempted to separate mortality after community‐acquired and nosocomial infections; however, it reported a low number of studies that differentiated. Pooled 30‐day mortality in 38 cohorts was 26% following any kind of infection (*N* = 15 722), which was comparable to the mortality found in our study (30%). Our study adds novel information to this comprehensive study by eliminating geographical and aetiological heterogenicity, whilst also distinguishing between community‐ and hospital‐acquired infections.

In conclusion, this study determined the risk of a first‐time community‐acquired serious systemic infection after being diagnosed with ALD cirrhosis. It advances our understanding of a serious condition in a vulnerable patient group, and the results are immediately useful for future trials on preventive measures [11]. The risk of community‐acquired serious systemic infections within the first year after diagnosis increased with increasing MELD‐Na score at the time of diagnosis. This finding may indicate that if preventive measures are to be included in the future clinical handling of ALD cirrhosis patients, we may want to consider the stages of cirrhosis and primarily target the patients with the most advanced cirrhosis. At last, our findings suggest that prophylaxis should initially target Enterobacterales, Staphylococci and Streptococci.

## Author Contributions

C.J.S., P.J. and K.S. designed the study. P.J. collected necessary approvals and access to clinical data. C.J.S., P.J., M.D. and M.J. performed statistical analysis. All authors contributed to interpreting results. C.J.S. drafted the manuscript, and all authors provided critical revisions and approved the final version of the manuscript for submission.

## Funding

The authors have nothing to report.

## Ethics Statement

According to Danish law, this study did not require ethics approval.

## Consent

Patient consent was waived by the Central Denmark Region, case number 1‐45‐70‐47‐23.

## Conflicts of Interest

P.J. has received honoraria from AstraZeneca outside of this work. All other authors have no conflicts of interest to disclose.

## Supporting information


**Table S1:** List of applied ICD‐10 discharge diagnosis codes.
**Figure S1:** Study design.
**Figure S2:** 90‐day mortality risk after sepsis or bacteraemia, *N* = 156.

## Data Availability

Danish law prohibits us from sharing the data, but code for analyses will be shared upon reasonable request.
