# Supplementary material for: Incidence and Mortality of Community‐Acquired Sepsis or Bacteraemia in Patients With Alcohol‐Related Cirrhosis
Source: Liver Int. 2026 Sep 15;46(10):e70881. doi: 10.1111/liv.70881 (PMC13575837; doi:10.1111/liv.70881)
Supplement: Supplementary file 1 [file LIV-46-0-s001.zip › liv70881-sup-0002-TableS1-FigureS1-S2@Revised Supplementary Data Liver International.docx]

# **Supplementary data**

## **Table S1.** List of applied ICD-10 discharge diagnosis codes.

| **Codes used to identify sepsis and septicaemia** |  | **No. of outcomes*** |
| --- | --- | --- |
| A40 | Streptococcal sepsis | 9 |
| A41 | Other sepsis | 66 |
| A021 | Salmonella sepsis | 0 |
| A207 | Septic plague | 0 |
| A227 | Anthrax sepsis | 0 |
| A267 | Erysipelothrix Rhusiopathiae sepsis | 0 |
| A282B | Yersinia sepsis | 0 |
| A327 | Listeria sepsis | 0 |
| A392A | Meningococcal sepsis | 0 |
| A427 | Actinomycosis sepsis | 0 |
| A548G | Gonococcal sepsis | 0 |
| A229B | Anthrax septicaemia | 0 |
| **Codes used to identify bacteraemia** |  |  |
| A392 | Acute Meningococcal bacteraemia | 0 |
| A393 | Chronic Meningococcal bacteraemia | 0 |
| A394 | Meningococcal bacteraemia (not specified) | 0 |
| A499A | Bacteraemia (not specified) | 2 |
| **Codes used to identify shock with bacteraemia or sepsis** |  |  |
| A483 | Toxic shock syndrome | 1 |
| R572 | Septic shock | 15 |

**A total of 93 patients were identified as having a first-time community-acquired serious systemic infection by a discharge diagnosis codes with or without a positive blood culture. 2 patients had a diagnosis code indicating bacteraemia (alongside positive blood cultures), whilst the other 91 codes indicated sepsis.*

## **Figure S1.** Study design.

## **Figure S2.** 90-day mortality risk after sepsis or bacteraemia, N=156.
