# Supplementary material for: Incidence and Mortality of Community‐Acquired Sepsis or Bacteraemia in Patients With Alcohol‐Related Cirrhosis
Source: Liver Int. 2026 Sep 15;46(10):e70881. doi: 10.1111/liv.70881 (PMC13575837; doi:10.1111/liv.70881)
Supplement: Supplementary file 1 [file LIV-46-0-s001.zip › liv70881-sup-0001-FigureS2@FigS2.pdf]

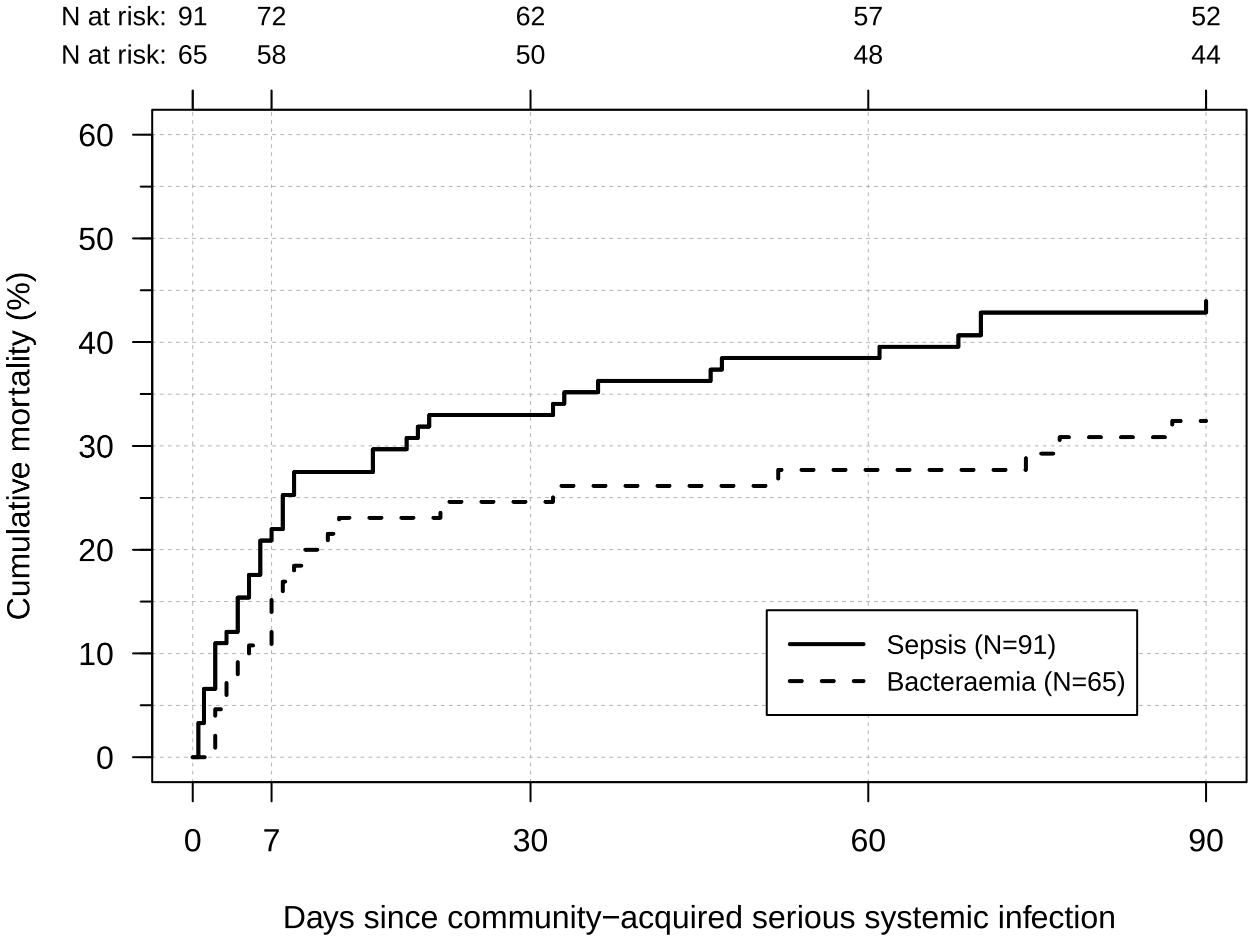

N at risk: 91 72 62 57 52
N at risk: 65 58 50 48 44
Cumulative mortality (%)
60
50
40
30
20
10
0
0
7
30
60
90
Days since community−acquired serious systemic infection
Sepsis (N=91)
Bacteraemia (N=65)
